# Case of Dislocation of the Humerus, Reduced at the End of Twenty-One Days

**Published:** 1838-10-10

**Authors:** J. F. Meigs

**Affiliations:** Resident Surgeon


### CLINICAL REPORTS.

### PENNSYLVANIA HOSPITAL.

*Case of Dislocation of the Humerus, reduced at the end of twenty-one days.*

[Reported by J. F. Meigs, M. D., Resident Surgeon.]

Admitted, September 19th, 1838, Elizabeth B., a domestic, æt. twenty-two, with a dislocation of the left humerus downwards into the axilla, caused by a fall down a stairway twenty-one days previously. She stated that she had been seen by a physician immediately after the accident, who, after slight manipulation, assured her that the bone was replaced. As her arm, however, remained useless, and she suffered much pain, she applied to Dr. Norris, on the 19th, who finding that the luxation remained unreduced, sent her to the hospital. At this time, the symptoms were those usually observed; the roundness of the shoulder was lost, the arm lengthened one inch, the head of the bone felt in the axilla, and the motions of the arm much impaired, with great pain. The patient having taken a grain and a half of tartar emetic, in divided doses, Dr. Norris proceeded at once to the reduction. A folded sheet was firmly applied by wet rollers to the arm, just above the elbow, to effect extension with the use of the pullies; another sheet was passed around the chest and secured to a staple, for counter-extension; and in order firmly to fix the scapula, a third band was applied over the acromion process, and given to assistants, who were directed to apply their force in a line obliquely downwards towards the opposite side. The apparatus being adjusted, a vein was opened, and extension kept up by two assistants, for the space of about fifteen minutes, at the end of which time all extension being suddenly removed and the arm brought close to the side, it was found that the roundness of the shoulder had returned, that the limb had become of the same length as the opposite one, and that the head of the bone could no longer be felt in the axilla. The patient being very much relaxed, the clavicle apparatus of the house, with the exception of the pad in the axilla, was applied, and she was placed in bed. No inflammation followed, and she left the house on the 3d of October with almost perfect use of her arm, slight stiffness merely remaining.

